# Proceedings of second Indian GAME conference, Mumbai, February, 2016

**DOI:** 10.3402/jecme.v5.32551

**Published:** 2016-10-13

**Authors:** Vaibhav Srivastava, Robin Stevenson, Shwetal Sanghvi

**Affiliations:** ^a^ Insignia Communications Pvt Ltd, Unit No. 202, Shreepal Complex, Suren Road, Opp Dipti Classic, Andheri (E), Mumbai, India; ^b^ Emeritus Professor of Respiratory Medicine, University of Glasgow, Glasgow, Scotland

**Keywords:** GAME India, CME system, Development, Recommendations, CME/CPD

## Abstract

The second Indian Global Alliance for Medical Education (GAME) conference on continuing medical education-continuing professional development (CME-CPD) was held in Mumbai in February 2016. The main aim of the meeting was to create a blueprint for further development of CME in India based on best practices from around the world. To that end, delegates had been invited from the USA, the UK and Australasia, who engaged in productive discussions with the major stakeholders of the CME community in India. The latter included clinicians, medical communications representatives and delegates from the pharmaceutical industry. The mandatory CME system already established in Maharashtra was described as an example, which could be exported to other states. The various types of accreditation were discussed, including provider and activity accreditation along with hybrid systems. Recommendations for future development were proposed from workshops comprising clinicians, industry representatives and medical communications agencies.

## Introduction: the winning game

Repeating last time's feat, the second Indian chapter of the regional conference of the Global Alliance for Medical Education (GAME), held in Mumbai at “The Leela” on 13 February 2016, was a runaway success, with big names from medical associations and councils, medcom companies and the industry gracing the event by their presence. Everyone was thrilled by the fact that the objectives of this august gathering were met. Together, these mighty minds attempted to define and structure the role of the healthcare industry in supporting and upgrading the Indian continuing medical education (CME) or continuing professional development (CPD) ecosystem. This report is a reflection of the grand happenings of this event and acts as a mouthpiece to spread good tidings about the earnest outcomes of the discussions that left the audience enthused.

## Prudently adopting global CME-CPD practices into the Indian CME ecosystem

Mr. Vaibhav Srivastava (Programme Director – Second GAME India Regional Conference, one of GAME's Board of Directors and Director of Insignia Communications) heartily welcomed the participants before setting up the agenda for the meeting and inviting the speakers to address the attendees. Going down memory lane, he recalled nostalgic moments of the first GAME Regional Conference that was conducted in India on 18 October 2014 and then went on to outline the objectives of the conference, namely, to create an Indian CME stakeholders’ forum with responsibility to upgrade the Indian CME ecosystem; to learn and understand from global CME practices and adopt the best in India; to debate, discuss and motivate Indian governing bodies to develop a uniform accreditation policy in India; to define and structure the role of the healthcare industry in supporting a healthy CME environment in India; and to explain the role of third parties (CME providers) in strengthening the Indian CME ecosystem. He concluded,

The aim of this conference is to provide a unique platform for all the stakeholders under a single roof. The CME system in India needs to be structured, and this will not be possible without the joint efforts of the key opinion leaders, state medical councils, Indian medical associations, CME providers, and the pharmaceutical industry. I am glad that the guests of honour who were present in the first event are here again.

## Roles and plans of the Medical Council of India for medical education

Dr. Vedprakash Mishra (Chairman – Academic Committee, MCI; Guest of Honour) methodically explained the role of the Medical Council of India (MCI) as a regulatory body. He pragmatically declared that standards are not set for cosmetic purposes, or just because a constitutional mandate exists. Dr. Mishra emphasised the need for uniform standards and central enactment, as he disclosed the current endeavours of the Council in trying to make doctors confident, concerned, competent and compliant. The minimum requirements for medical institutions have been chalked out in terms of infrastructure, personnel and approaches. Furthermore, the curriculum design and updating process are also in place with well-developed teaching and learning strategies. The Council also has a major role to play in postgraduate education.

He added that a competency-based integrated curriculum has been advanced, which is learner-centric, technically as well as technologically sound, and replete with assessment modules. Such a curriculum would be current plus futuristic, with the imperatives being in relation to the happenings in the past 20 years. He proudly announced, “India is the largest producer of trained healthcare manpower; and according to the World Health Organisation, India will lead in global healthcare delivery by the year 2050. Thus, Indian graduates will have to shoulder these responsibilities worldwide.” He mentioned that the need of the hour is specialty discussions that are timely and well-reasoned. However, the time frame of an update has to be taken into consideration. Shared experiences need to be put across early for the benefit of the medical fraternity. Unfortunately, a gestational period of nearly 5 years has been observed between medical updates, while the Bachelor of Medicine and Bachelor of Surgery (MBBS) course itself is of 4.5 years, which is bound to leave an educational lag. This limitation is not manual, rather procedural. Hence, he strongly felt that academic updates should not be at the mercy of the Government of India; and even if the government's approval is necessary, the matter should be cleared within 90 days. He appreciated the fact that GAME has accepted the onus of helping to improve the CME ecosystem in India and declared that all the stakeholders together can certainly make a positive difference.

## Role of the OPPI in supporting the Indian CME ecosystem

Mr. Sudarshan Jain (Vice-President – Organisation of Pharmaceutical Producers of India (OPPI); Managing Director, Healthcare Solutions – Abbott Healthcare) started his presentation with some appalling statistics. He revealed that with the number of specialists being limited and that of generalists being high in India, there is a need for interventions that facilitate an education cascade from generalists to specialists through the agency of CME.

He added that by the end of 2020, the disease-related mortality in developing countries is expected to mimic that of the developed nations. Thus, ischaemic heart disease, diabetes mellitus and depressive disorders will be the major killers, while gastrointestinal diseases with diarrhoea and respiratory conditions such as tuberculosis and chronic obstructive pulmonary disease will no longer top the charts. Mr. Jain advised,

There is a need to build a patient funnel. The prevalence of diseases can be reduced by driving patient awareness using multiple touchpoints. Accessibility to a larger pool of medical practitioners will help to reach more patients for better diagnosis and appropriate treatment. Facilitation of guideline development and dissemination will definitely raise treatment standards.

He affirmed that the pharmaceutical industry will play a key role in establishing the CME ecosystem by identifying unmet educational needs and collaborating to develop and enable compliant, transparent and standardised CME. According to him, the focus areas of OPPI include continuing dialogue with relevant stakeholders, engaging actively in creating an ecosystem for knowledge sharing with value additions and facilitating interactions between the industry and academia.

## Game and its goals: a review

A veteran in the global pharmaceutical and medical communications industry, Ms. Lisa Sullivan (President – GAME) shared details about the Alliance. Founded in 1995, this not-for-profit international organisation has over 150 members from diverse geographies backgrounds, namely, academics, health personnel, society members, accreditors and people from the industry. Each of these stakeholders is dedicated to advancing innovation and collaboration in continuing medical education and professional development across the world.

Spelling out the goals and activities of the Alliance, she said,

GAME facilitates the link between continuing professional development, undergraduate medical education, and postgraduate medical education – otherwise known as the medical education continuum. It enables CME or CPD professionals globally to leverage the science of learning and change, while helping their learners (healthcare professionals) to close knowledge and practice gaps, so as ultimately to lead to improved patient care and better clinical outcomes. It creates a network of CME or CPD professionals to collaborate, innovate, and elevate the standards of need-based medical education worldwide.

She was proud that GAME had successfully launched its first Indian Regional Conference in October 2014, first South American Regional Conference in November 2014 and first Canadian Regional Conference in May 2015. The second Indian Regional Conference is underway, and the second Canadian Regional Conference is currently under discussion. The Alliance is also planning to hold a regional conference for the first time in the United Arab Emirates (UAE). The second annual conference in partnership with the Association for Medical Education in Europe (AMEE) will be conducted in August 2016 in Barcelona, and a collaborative relationship with the Asia Pacific Medical Education Conference (APMEC) will begin from next year onwards. GAME has also partnered with the Association for Continuing Education for Health Professionals (ACEhp) to conduct joint sessions and was involved in two sessions at the European CME forum in November 2015. The addition of Special Interest Groups (SIG) has also got everyone excited. These groups include members of the International Pharmaceutical Alliance for Continuing Medical Education (iPACME), patient engagement groups, and individuals from medical education companies and specialty or learned societies.

## The success story of making CME mandatory in Maharashtra

The success story of Maharashtra's mandatory CME was shared by Dr. Shivkumar Utture (Executive Member and Chairman, Finance Committee – Maharashtra Medical Council (MMC); Finance Secretary – IMA, Maharashtra State), who reminded the audience of two important statements from the code of ethics, which required a physician to affiliate with associations and societies of allopathic medical professions and participate in professional meetings as part of CME programmes, for at least 30 hours every 5 years. He implied that once the regulatory bodies and CME providers come together, they can chalk out the programme. Patients form the core of the health system; therefore, a patient-centric approach is important.

He reasoned, “Once a person gets a degree, if he or she does not upgrade his or her knowledge, he or she will not be able to provide the best services to patients. Hence, the decision to link CME credit points to the re-registration process every 5 years was taken.” The legalities took nearly 5 years to complete, and everything was eventually put down in black and white. Dr. Utture asked the participants to visit the MMC website and go through the 20 chapters that it contains. The website, he said, is well developed and has multiple functionalities such as a payment gateway, links for the upload and download of forms, and dedicated pages for each of its members. Speaking about the modalities for accreditation, he discussed the who, how and what of the process and explained how the MMC has taken several national and state-level organisations under its ambit and grants them accreditation if they fulfil all the requirements. It sends inspectors at regular intervals to monitor the activities of these organisations. These inspectors are mostly teachers from government medical colleges, who are appointed by the IMA to attend the CME event and to give feedback. The accreditation continues on the basis of these reports.

## Role of the IMA in the Indian CME ecosystem

Dr. Jayesh Lele (President – IMA, Maharashtra State; Honorary Joint Secretary – IMA CGP, Maharashtra State) added to the details provided by Dr. Utture, giving more information about IMA and its role in the CME ecosystem of Maharashtra. He revealed, “The IMA has about 270,000 allopathic practitioners across 1,800 branches in India, and IMA Maharashtra has 204 branches with 37,000 members across Maharashtra. Due to this strong network, the Association has the ability to conduct any medical programme or CME event.” All the IMA branches in Maharashtra conduct regular CME activities for their members. In the past, national programmes such as the “AIDS awareness campaign” from the Gates Foundation have been conducted by the Association. It also ensures that there are regular CME updates whenever issues like swine flu, Zika virus infection or dengue crop up. These CME activities are conducted jointly with municipal corporations or local health authorities. It also facilitates the observance of world health days for conditions such as epilepsy, acquired immune deficiency syndrome (AIDS), diabetes, kidney disorders and heart disease.

He said that the IMA is actively involved in numerous patient benefit programmes, such as awareness campaigns and medical camps for medical check-ups, blood donation and vaccination. Village adoption programmes have also been started. The MMC formulated CME Guidelines in 2014, and all IMA branches and some specialty medical associations were given accreditation after scrutiny. The speaker accreditation process has also been formulated and is available online.

## Need for a uniform accreditation system and the challenges associated with it

The discussion on the need for a uniform accreditation system and its associated challenges included as panellists Dr. Murugunathan (Dean – Indian College of Physicians; President – Hypertension Society of India), Dr. Girish Tyagi (Registrar – Delhi Medical Council), Dr. Utture, Dr. Jamshed Dalal (Director – Cardiac Services, Kokilaben Dhirubhai Ambani Hospital, Mumbai), Dr. Hrishikesh Pai (President – Indian Society of Assisted Reproduction) and Dr. Vibhore Awasthy (CME Director – Insignia Communications) who was the moderator of the session. Dr. Dalal strongly felt that the current CME scenario was full of foul play, with a lot of misuse taking place, just for the sake of credit points. He felt that this trend should be discouraged. Dr. Pai echoed the same sentiment, but added that the concept of CME needs to be made acceptable. A major challenge pointed out by Dr. Murugunathan was that the credit points vary with different councils for the same CME. Dr. Tyagi agreed with the other panellists and expressed the need for a uniform consensus and guidelines that should be made mandatory. Dr. Vibhore recommended the use of online CME and post-CME assessments to ensure that the doctors were certainly learning something. The panellists unanimously declared that the Maharashtra model needed to be replicated in all the other Indian states. They also felt that attending some CME activities needs to be made mandatory, while others can be attended by choice.

## What the industry thinks about CME challenges and opportunities

Mr. Sanjiv Navangul (Managing Director – Janssen India) explained the challenges and opportunities related to CME from the industry perspective. He remarked,

Most healthcare providers feel that pharma companies push for attendance and use CME as a medium to promote company products. The perception of pharma regulators is that these companies perpetuate treatment for conditions, which need no deliberate interventions; and spend a lot of money that could have been used otherwise, thereby reducing the cost of drugs. The medical regulators, on the other hand, sense that the Industry has only commercial interests, which conflicts with the interests of the different stakeholders. They believe that the goals of the healthcare providers and pharma companies are not the same; and hence, do not entertain accreditation requests by the latter.

He stated that there are no defined guidelines, standardised protocols and regulations for contribution from the industry. Moreover, there is a lack of transparency in the processes. Very few web-based CME activities are accredited, and print-based CME carries no credit points at all. Attempts by the industry to provide doctors with access to books or journals are considered to be marketing strategies. Hence, a principled and balanced approach is required with a collaboration between the industry and academia. Strict codes of conduct need to be adhered to, with the content being free from any sponsor inputs. The delivery of educational programmes should be improved and must include non-traditional learning formats, such as e-learning modules. Above all, the healthcare providers should perceive the value of continuing education.

## Role of the different stakeholders in the global CME ecosystem

The role of the different stakeholders in the global CME ecosystem was discussed by international faculty members, namely, Ms. Lisa Sullivan, Ms. Maureen Doyle-Scharff (Immediate Past President – GAME), Ms. Aimee Brinzer (Managing Director – Wilmington Healthcare) and Professor Robin Stevenson (Glasgow University; Editor – *Journal of European CME*) who moderated the session. Dr. Narendra Sainani (Board Member – Delhi Medical Council) and Dr. Banshi Saboo (Joint Secretary – RSSDI) were also esteemed panellists in this discussion. Speaking about the US scenario, Ms. Doyle-Scharff specified that all the CME providers there had to be accredited by the ACCME. The current trend involves joint accreditations, where a single CME module can be tweaked to cater to the needs of different target audiences. Unlike in India, where the medical council decides on the credit points for a CME activity, the provider does so in the USA. Ms. Sullivan revealed that a similar system was also followed in Australia. Professor Stevenson said that in Europe a hybrid system was being developed in which major CME providers are accredited, whereas in the case of minor CME providers, individual CME activities are accredited.

Ms. Brinzer spoke about the current system in the UK, where the focus is on appraisal. This is an important tool for monitoring the standards of patient care. Appraisal incorporates certain elements such as reflections of the clinician's practice, patient feedbacks, peer reviews and case studies in addition to review of CME activities. Thus, there are representatives for every section. Dr. Sainani claimed that such a process would not be feasible in India, considering logistics. Furthermore, making CME mandatory in all parts of India is difficult, as doctors residing and practising in remote areas will find it difficult to be physically present. Dr. Saboo added here that several hospitals in India do conduct CME programmes for their staff; however, these are often not accredited by any society. Professor Stevenson pointed out that “the patient” was not mentioned as a stakeholder at all, the truth being that all medical CME should be workplace specific, more effectively to change the practice patterns of doctors. He also felt that the industry should, ethically speaking, operate at arm's length.

## Contribution of iPACME to the CME world

Ms. Maureen Doyle-Scharff updated the audience about the contribution of iPACME to the global CME ecosystem. She revealed that the iPACME was established in 2010 and includes employees of global and regional pharmaceutical companies. The body is responsible for providing medical education grants and developing CME programmes. It provides a forum for members interested in advancing innovations in CME/CPD and allows for exchange and sharing of best practices (appropriately allowed within the law) between industry representatives actively engaged in the CME/CPD enterprise.

Its key initiatives have been providing an online forum to discuss key issues with the group, to network, and to ask and answer questions, creating a global lexicon for medical education, developing a medical education Wiki (establishing a common language for CME/CE/CPD professionals around the world) and forming a subcommittee in 2014 to develop a guidance document to set standards and processes for the industry in Europe. She declared,

The pharmaceutical industry wants to be recognised as a valued and trusted partner for the provision of high quality education that complements existing activities and meets the educational needs of healthcare providers in improving patient care.

## Way forward to building an ideal CME ecosystem in India

This brainstorming session, which was moderated by Mr. Lawrence Sherman (Senior Vice-President, Educational Strategy – TOPEC Global), had as panellists Dr. Rajesh Upadhyay (Past President – API), Dr. Suresh Vashisth (President – ASI), Ms. Swati Dalal (Director – Commercial Operations, Abbott Healthcare) and Mr. Vaibhav Srivastava. Dr. Suresh suggested that all CME should be both evidence-based and needs-based, after knowing the requirements of the audience. Dr. Rajesh was in agreement with him and said that India is a very diverse country, and the need of the hour is to reach out to the rural areas which constitute 70% of its population. Thus, CME has to be relevant to the learners. Ms. Dalal added that the entire process needs to be simplified, and a needs gap analysis should ideally be done before developing any programme. Mr. Srivastava also believed that the processes should be reduced to the minimum, with the learning objectives being clearly defined and the right set of assessments being used before and after such endeavours. Speaking about the nature of the faculty, Mr. Sherman admitted that a great scientist may not be a very good educator. So, when accrediting a speaker, this must also be taken into consideration. When the discussion shifted to defining CME better, Dr. Suresh joked that it could be definitely not stand for cheating, meeting and eating.

## Multidisciplinary workshop discussions on future development of CME ecosystem

Three workshops comprising clinicians, representatives from industry and delegates from medical communications agencies discussed in detail the topics already presented to the meeting. They were charged with developing recommendations for the future shaping of Indian CME. A composite report of their deliberations is presented below.

### 1. On CME accreditation process

### a. Is there the need for a uniform CME accreditation policy across India (by all states)?

***Recommendation: Yes, there has to be a uniform CME accreditation process throughout the nation**.*

A single central governing body with MCI or a pivotal central body either controlled by MCI or a part of MCI should be instituted to lay down the rules and regulations for CME accreditation throughout the country. These rules should be in discussion with various state councils and approved by all the state medical councils, thereby leaving no space for any ambiguity.

### b. Is there a need to recognise interstate CME credit points and bring in MCI as the National Accreditation Body to accredit CME-CPD applicable to multiple states HCPs?

***Recommendation: Yes, an interstate CME accreditation body attended by a healthcare practitioner (HCP) should be recognised for an approved credit system as determined by the central governing body**.*

A single central governing body like MCI or a pivotal central body either controlled by MCI or a part of MCI should state in the book of rules and regulations the credit points to be allocated for interstate CME as in consideration with the specific state medical council.

### 2. On CME providers

### a. Should there be guidelines for CME providers’ selection criteria (like USA)?

***Recommendation: Yes, there should be guidelines for recognition of any medical institutions, medical colleges and third-party medical communications agencies as CME providers. This needs to be addressed by the state medical council**.*

Recognition of a CME provider should be clearly mentioned in the rules and regulations set forth by the central governing body, and the CME providers need to adhere to these rules. A provider must engage in education rather than marketing.

### b. Should all the competent bodies such as national/regional/local medical associations, medical colleges and third-party CME providers in India be recognised?

***Recommendation: Yes, all the competent medical bodies such as national/regional/local medical associations, medical colleges and third-party agencies who meet the criteria set by the central governing body should be recognised as CME providers**.*

The pharmaceutical industry should act as an enabler rather than a provider, and communications agencies should act as facilitators rather than providers.

### 3. On CME execution process

### a. Is there a need that each accredited CME-CPD system follows a minimum process guideline (need gap, learning objective, right set of learner and outcome analysis)?

***Recommendation: Yes, there is a need that each accredited CME-CPD system follows a minimum process like meeting the needs gap, learning objectives and the outcomes**.*

The governing body should clearly have a mandate for addressing the needs gap of CME-CPD in order to take the programme to the right audience, to address the correct learning objectives for the intended audience as well as to have an outcome analysis.

### b. Should there be a separate CME department within CME providers organisation/associations or an outsourced competent agency to accomplish the above task.

***Recommendation: Yes, a separate CME department within CME providers/associations or a third-party competent CME facilitator that meets the criteria set by the central governing body is needed**.*

The MCI should have a separate CME department within CME providers/associations or a third-party competent CME facilitator. This would be in association with the state councils.

### 4. On CME categorisation

Whether we should define and recognise different types of CME, that is:

- Independent medical education (IME)
- Company-driven, product-specific education programme
- Company-initiated professional development/medical disease education
- Collaborative partnership programme ( industry and HCOs)

***Recommendation: No, there should be no categorisation of CME. Any CME should meet the criteria of learning, thereby assuring better patient outcomes**.*

Company-driven or product-specific CME should not be accredited.

### 5. On CME-CPD medium/mode

### Should we recognise all modes of learning – live/online/print or not?

***Recommendation: Yes, all modes of delivering CME-CPD should be recognised (live/online/print-based learning)**.*

### 6. On recognising international accredited CME

### Should we recognise ACCME/EACCME/RCP-accredited CME (which are relevant for Indian HCPs) or not?

***Recommendation: Yes, ACCME/EACCME/RCP-accredited CME can be recognised for accreditation, provided that these are relevant to Indian medical education and can be reciprocated and reproduced in the Indian clinical setting**.*

### 7. On CME funding

### Should there be proper guidelines for appropriate funding declaration on all relevant documents and announcements?

***Recommendation: Yes, funding declaration is required with proper guidelines defined by the central governing body**.*

